# Generation of a H9 Clonal Cell Line With Inducible Expression of NUP98-KDM5A Fusion Gene in the AAVS1 Safe Harbor Locus

**DOI:** 10.3389/fcell.2022.846092

**Published:** 2022-06-01

**Authors:** Joan Domingo-Reinés, Gonzalo Martínez-Navajas, Rosa Montes, Mar Lamolda, Iris Simón, Julio Castaño, Rosa Ríos-Pelegrina, Javier Luis Lopez-Hidalgo, Raimundo García del Moral, Juan A. Marchal, Pedro J. Real, Verónica Ramos-Mejía

**Affiliations:** ^1^ GENYO, Centre for Genomics and Oncological Research Pfizer-University of Granada-Andalusian Regional Government, Granada, Spain; ^2^ Faculty of Science, Department of Biochemistry and Molecular Biology I, University of Granada, Granada, Spain; ^3^ Faculty of Sciences, Department of Cell Biology, University of Granada, Granada, Spain; ^4^ Advanced Cell Therapy Service, Banc de Sang I Teixits, Edifici Dr. Frederic Duran I Jordà, Barcelona, Spain; ^5^ Unidad de Anatomía Patológica, Hospital Universitario Clínico San Cecilio, Granada, Spain; ^6^ Instituto de Investigación Biosanitaria Ibs.GRANADA, Granada, Spain; ^7^ Biopathology and Regenerative Medicine Institute (IBIMER), Centre for Biomedical Research, University of Granada, Granada, Spain; ^8^ Faculty of Medicine, Department of Human Anatomy and Embryology, University of Granada, Granada, Spain; ^9^ Excellence Research Unit “Modeling Nature” (MNat), University of Granada, Granada, Spain

**Keywords:** acute myeliod leukemia, pediatric, disease model, human stem cell, hematopoieisis

## Abstract

Pediatric acute myeloid leukemia (AML) is a rare and heterogeneous disease that remains the major cause of mortality in children with leukemia. To improve the outcome of pediatric AML we need to gain knowledge on the biological bases of this disease. NUP98-KDM5A (NK5A) fusion protein is present in a particular subgroup of young pediatric patients with poor outcome. We report the generation and characterization of human Embryonic Stem Cell (hESC) clonal lines with inducible expression of NK5A. Temporal control of NK5A expression during hematopoietic differentiation from hESC will be critical for elucidating its participation during the leukemogenic process.

## Introduction

Acute Myeloid Leukemia (AML) contributes to 20% of acute leukemia cases in children and, although the overall survival has improved over the past years, still around 50% of the pediatric patients will experience relapse (de Rooij et al., 2015; AplencMeshinchi et al., 2020). Pediatric AML is a heterogeneous group of blood disorders characterized by different genetic abnormalities that are frequently originated during embryonic development in a still unknown cell type(s) (Cazzola et al., 2020).

To overcome the suboptimal outcomes of AML in children, we need to generate relevant research models to broaden our knowledge on pediatric AML and develop new treatment approaches. Leukemia cells lines and animal models have been essential for a better understanding of leukemia, but usually fail recapitulating some developmental processes of tumor formation in human (Milan et al., 2019). Human pluripotent stem cells (hPSC) have the capability of self-renewal and the potential to differentiate into any cell type, giving the possibility to study embryonic hematopoietic development obtaining different types of hematopoietic progenitor cells (Slukvin, 2013). A critical aspect for the study of pediatric AML is the expression of the driver genetic alteration in the proper cell type during hematopoietic development. Thus, controlling the timing of expression of the oncogene will allow us to explore which cell populations are more suitable for leukemic transformation.

The NUP98-KDM5A chimeric oncoprotein is exclusively found in young pediatric AML patients with high risk and poor prognosis.^6^ Here, we describe the generation and characterization of the H9 iNK5A cell line, a human Embryonic Stem Cell (hESC) model with doxycycline-regulated expression of NUP98-KDM5A fusion protein, allowing the temporal control of NUP98-KDM5A during hematopoietic differentiation from hESC, to recapitulate and study the leukemogenic process that occurs in these patients during embryonic development.

## Methods

### Generation of the Inducible Cell Line Using CRISPR/Cas9 Technology

The cDNA of the NUP98-KDM5A was obtained commercially fusing the first 1542 nucleotides of NUP98 and the 4467–5070 nucleotides of KDM5A, adding at the beginning the sequence of a 3x-FLAG tag at TWIST Bioscience (San Francisco, California, United States). The cDNA was subcloned by standard procedures from a pTWIST to the pTRE3G plasmid. To edit the H9 cell line, 200.000 single cells were electroporated with 100 pmol Cas9 nuclease (IDT–Integrated DNA technologies, Coralville, Iowa, United States), 120 pmol gRNA against AAVS1 intron 1 (5′-GGG​GCC​ACT​AGG​GAC​AGG​AT-3′, IDT–Integrated DNA technologies) and 5 μg of linearized donor vector. The edition was performed following the previous procedure (Milan et al., 2019). Electroporation was performed using the program A-023 in a Nucleofector^TM^ 2b Device (LONZA, Basel, Switzerland) and the Human Stem Cell Nucleofector^TM^ kit 1. Cells were selected with 100 μg/ml of G-418 (Sigma-Aldrich, Saint Louis, Missouri, United States).

### Cell Culture

Cells were grown in feeder-free conditions using Essential 8 Medium (E8) over Matrigel® Matrix (Corning; Corning, New York, United States) diluted 1:40. Passages were performed every 3–5 days at a ratio of 1:6–1:10 using PBS-EDTA (0.5 μM) with Y-27632 2HCl (10 μM, Selleckchem, Houston, Texas, United States). The conditions used were 37°C, 5% CO_2_ and 20% O_2_ in a humid incubator.

### PCR and RT-PCR Analysis

Genomic DNA was extracted with the NucleoSpin Tissue kit (Machery-Nagel; Düren, Germany). Total RNA was isolated with the *Illustra RNAspin Mini kit* (GE Healthcare; Chicago, Illinois, United States) and cDNA was retrotranscribed with the *Transcription First Strand cDNA synthesis kit* (Roche; Basel, Switzerland). RT-PCR analysis for the quantification of insertions and the expression of genes was performed using *iTaq Universal SYBR Green Supermix kit* (Biorad; Hercules, California, United States) in a QuantStudio 6 Flex Real-Time PCR Systems (Applied Biosystems; Waltham, Massachusetts, United States). Primer sequences used are shown in Supplementary Table S1.

### Western Blot

Total protein was extracted with RIPA buffer (Sigma-Aldrich) containing protease inhibitor cocktail (Roche) and phosphatase inhibitors cocktail 2 and 3 (Sigma-Aldrich). Cell lysates were separated using SDS-polyacrylamide gels and transferred to polyvinylidene fluoride (PVDF) membranes. Protein was detected using the Odyssey Infrared Imaging System (Li-cor Biosciences; Lincoln, Nebraska, United States). To detect the fusion protein was used the anti-KDM5A antibody (Abcam; Cambridge, United Kingdom, ab70892) and the anti-FLAG antibody (Sigma, F3165).

### Flow Cytometry Analysis

Single cell suspensions were stained for Tra-1–60, Tra-1-81 and SSEA4. A relevant isotype-matched antibody was used as a negative control. Live cells were identified by 7-aminoactinomycin D exclusion and were analysed using a FACS Verse (BD Bioscience; San Jose, California, United States) and FlowJo X10 Software (BD Bioscience). Primary antibodies are included in Supplementary Table S1.

### Embryo Body Differentiation Assay

*Matrigel* 1:6 (diluted in KO-DMEM, Thermofisher; Waltham, Massachusetts, United States) was added to the culture to obtain 3D colonies. For EBs formation, hPSC colonies were gently scraped off the flask, resuspended into Essential 6 medium (E6) and plated over low attachment 6-well plates (Corning). The cells were cultured with or without 2 μg/ml of doxycycline during 21 days. The EBs were centrifuged, fixed, embedded in paraffin and sectioned for histological and immunochemical analysis. Diaminobenzidine was used as the chromogen. The protein expression of CKAE1AE3 (Endoderm), Vimentin (Mesoderm) and TUBB3 (Ectoderm) were analyzed by Immunohistochemistry.

### Hematopoietic Differentiation

To differentiate hESCs into hematopoietic progenitors we used the STEMdiff™ Hematopoietic Kit (Stem cell technologies, Cat#05310; Vancouver, Canada) following manufacturer´s instructions. Briefly, we seeded from 16 to 40 clumps of cells into 12-well plates and during the first 3 days we used Medium A and then we switched to Medium B until day 12. For doxycycline treatment, we added the drug at 0.5, 1 or 2 μg/ml to the differentiation media starting at day 3 or 5 of the hematopoietic differentiation. At day 12 the floating cells were harvested, stained for flow cytometry and pelleted for RT-PCR analysis.

### Immunofluorescence

Samples were fixed with 4% paraformaldehyde (Sigma-Aldrich). The samples were blocked and permeabilized with 5% BSA/PBS solution (Sigma-Aldrich) and 0.3% of triton x-100 (Sigma-Aldrich). The staining was performed overnight using a 5% BSA/PBS solution and the α-POU5F1 antibody. The images were taken with a Zeiss Axio imager A.1 microscope (Zeiss; Oberkochen, Germany) and analysed with the FIJI software. Primary antibodies are included in Supplementary Table S1.

### Karyotyping

Chromosomal analysis of cell lines was accomplished by GTG-banding analysis by the Andalusian Public Health System Biobank (Spain), according to the International System Cytogenetics Nomenclature recommendations. 20 metaphases were counted per analysis.

### Short Tandem Repeat Polymorphism Profiling

Genetic identity was determined with the *AmpFLSTR® Identifiler® Plus kit* (Applied Biosystems^TM^).

## Results

### Generation of an Inducible System for NUP98-KDM5A Fusion Gene Using Human Pluripotent Stem Cells

**FIGURE 1:**
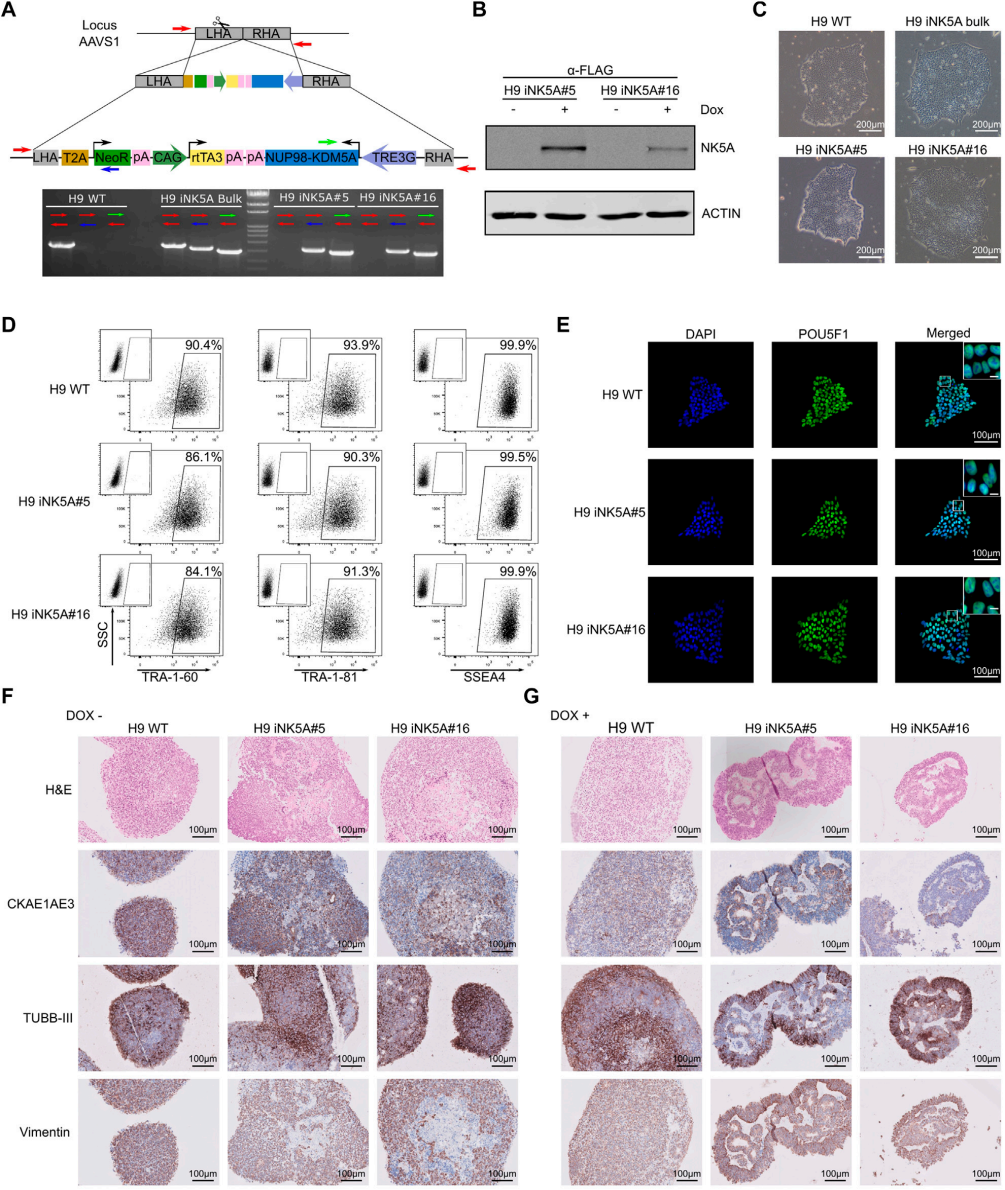
Generation and characterization of an inducible cell line for NUP98-KDM5A. **(A)** Scheme of the construction used for genome edition and homologous recombination into the AAVS1 locus. Arrows indicate primers used to detect the vector integration. **(B)** Western blot analysis of H9 iNK5A#5 and #16 clonal lines after doxycycline treatment to detect NUP98-KDM5A fusion protein with the anti-FLAG antibody. We used ACTIN as a loading control. **(C)** Bright field images showing the morphology of the colonies, which display a typical round shape with small, tightly packed cells in all hESC lines. Scale bar = 200 μm. **(D)** Expression of pluripotency-associated markers TRA-1-60, TRA-1-81, and SSEA4 at protein level by flow cytometry in H9 WT, H9 iNK5A #5 and #16 lines. Isotype control antibody at the upper left corner of each plot. **(E)** Immunofluorescence against POU5F1 showing typical nuclear staining. DAPI represents DNA with nuclear staining. Scale bar = 100 μm. White square shows a detail of nuclear staining. Scale bar = 10 μm. **(F)** Hematoxylin and eosin, CK AE1 AE3, TUBB-III and Vimentin staining in embryoid bodies (EBs) sections of H9 WT and H9 iNK5A #5 and #16 clones. EBs were differentiated during 21 days without doxycycline. Scale bar = 100 μm. **(G)** Hematoxylin and eosin, CK AE1 AE3, TUBB-III and Vimentin staining in embryoid bodies (EBs) sections of H9 WT and iNK5A #5 and #16. EBs were differentiated during 21 days with doxycycline. Scale bar = 100 μm.

We edited the H9 cell line (WA09, Wicell; Madison, Wisconsin, United States) at passage 40 using the CRISPR/Cas9 system to insert the NUP98-KDM5A (NK5A) coding sequence controlled by a doxycycline-inducible cassette in the genomic AAVS1 safe harbor locus. A vector containing both, the rTetR activator under CAG promoter and the Tetracycline Response Element (TRE) promoter driving the expression of FLAG-tagged NK5A, was inserted in the intron 1 of AAVS1 locus by Homology Directed Repair mechanism using an AAVS1 specific sgRNA together with the Cas9 nuclease (Figure 1A). We designed specific PCR genotyping primers (red, blue, and green arrows) to determine AAVS1 locus specific targeting and homozygosity. The H9 WT control only amplified the PCR product corresponding to the AAVS1 unedited locus (red primers), while the original transgenic H9 iNK5A line (H9 iNK5A bulk) produced products with all primer combination, indicating a mixture of cellular genotypes. The derived clonal cell lines H9 iNK5A#5 and H9 iNK5A#16 only amplified products using the primers targeting the insert (blue and green primers), indicating homozygosity (Figure 1A). We determined the plasmid copy number insertion by qPCR using a standard curve generated with known amounts of plasmid DNA. The H9 iNK5A bulk culture has 0.31 copies/cell, while the two clones have 2 inserts/cell (Supplementary Figure S1A). We also confirmed the correct genomic integration of the vector within the H9 iNK5A#5 and H9 iNK5A#16 clones by sequencing the fully NUP98-KDM5A cDNA sequence together with both gDNA homology arms (Supplementary Table S1). Furthermore, we analyzed the potential *in silico* off-targets of our sgRNA (RNF4, RHOT2, FAIM2, RPL8, BTNL8, and MYBL2) (Castaño et al., 2017) (Supplementary Table S1) by sanger sequencing, proving indels absence in their sequences.

Functionally, we validated the doxycycline inducible NK5A expression in the H9 iNK5A clonal cell lines by the addition of 2 μg/ml of doxycycline to the media during 48 h. By RT-PCR analysis we observed a very low leaking RNA expression of the fusion gene in the cells when grown without doxycycline, while the addition of doxycycline induced the NK5A expression more than 750 times (Supplementary Figure S1B). Nonetheless, by western blot analysis we detected the expression of the NK5A fusion protein only in cells treated with doxycycline, either using an anti-FLAG antibody (Figure 1B) and with an anti-KDM5A antibody that recognizes the endogenous form of KDM5A (196 kDa) and also detect the NK5A fusion protein at the expected molecular weight (76 kDa) (Supplementary Figure S1C). The H9 WT and H9 iNK5A bulk and clonal cell lines displayed the typical pluripotent morphology of compact colonies of small cells with prominent nucleus (Figure 1C). The H9 WT and H9 iNK5A clonal cell lines expressed similar levels of the pluripotency transcription factors POU5F1, SOX2, NANOG and REX1 (Supplementary Figure S1D). In H9 iNK5A clonal cell lines we also confirmed the expression of pluripotent surface markers TRA-1-60, TRA-1-81 and SSEA4 by flow cytometry (Figure 1D), and the expression of POU5F1 at the single cell level by immunofluorescence (Figure 1E). Finally, the short tandem repeat (STR) analysis corroborated the identity of the H9 iNK5A clonal cell lines (Supplementary Figure S1E) that also presented a normal female karyotype (Supplementary Figure S1F).

### The Expression of NUP98-KDM5A Affects the Cell Differentiation Process in Embryoid Bodies.

We tested the functionality of the doxycycline inducible system during a differentiation process using the embryoid body (EBs) formation assay. We formed EBs from H9 WT and the H9 iNK5A clonal cell lines and from day one onwards we treated one group with 2 μg/ml of doxycycline, while we maintained untreated a second group. After 21 days of EB formation, the induction of the NK5A fusion protein was checked by western blot and the fusion protein was detected only in iNK5A#5 and #16 EBs cultured in presence of doxycycline (Supplementary Figure S1G). As compared with EBs derived from H9 WT and H9 iNK5A cultured without doxycycline, the induction of NK5A fusion protein produced smaller EBs with visible secondary structures on the inside (Supplementary Figure S1H). We examined the histological structure of embryoid bodies using hematoxylin and eosin (H&E) staining and in all the EBs cultured without doxycycline we observed the predominance of small cells with homogenous chromatin, scant cytoplasm, and variably distinct cell borders (Figure 1F). In contrast, iNK5A#5 and #16 EBs treated with doxycycline had a more heterogeneous cell constitution with different sizes and morphologies, with the presence of pseudoepithelial-like pattern, frequent rosette-like structures, and areas of fibrous stroma (Figure 1G). Immunohistochemistry analysis demonstrated the pluripotent capacity of all cell lines as we detected the presence of markers for the three germ layers (cytokeratin AE1/AE3 (CK AE1 AE3) for endoderm, tubulin beta-3 chain (TUBB-III) for ectoderm and vimentin for mesoderm) in the spontaneously differentiated cells inside all EBs. The EBs from H9 WT and the iNK5A#5 and #16 lines in the absence of doxycycline mainly presented cells co-expressing two or more markers, indicating a primitive stage of differentiation (Figure 1F). In contrast, EBs from the iNK5A clonal cell lines treated with doxycycline produced more differentiated cells, with recognizable structures and differential staining distribution of the germ layer markers. While the CKAE1AE3 and vimentin staining were prevalent on the basal area of pseudoepithelial-like and rosette-like regions, the TUBB-III staining was present preferentially on the external regions of the EBs (Figure 1G).

### Effect of Inducible NK5A Expression on Hematopoietic Differentiation of H9 iNK5A Cells

**FIGURE 2:**
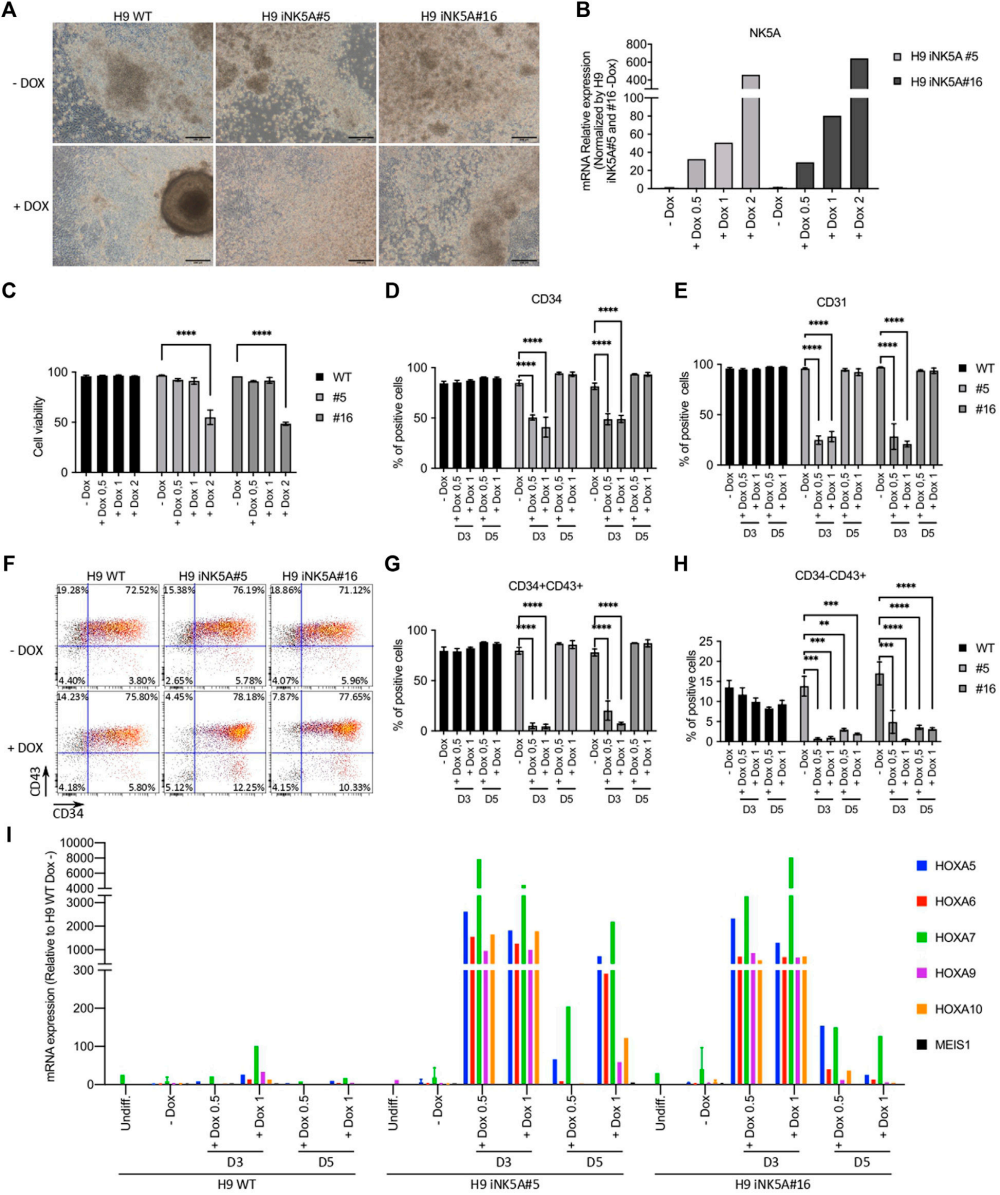
Induction of NUP98-KDM5A expression during hematopoietic differentiation. **(A)** Bright field microscopy pictures of the hematopoietic differentiation cultures at day 12 from H9 WT, H9iNK5A#5 and #16 with (+DOX) or without (-DOX) doxycycline treatment. Scale bar = 200 μm. **(B)** RT-PCR analysis of NUP98-KDM5A expression after induction with 0.5, 1 or 2 μg/ml of doxycycline starting at day 3 of differentiation. GAPDH, rRNA 18S, RPL7, and B2M were used as housekeeping control genes. **(C)** Flow cytometry analysis at day 12 of the cell viability (%7AAD negative cells) after NK5A induction with 0.5, 1 or 2 μg/ml of doxycycline starting at day 3 of differentiation. **(D)** Flow cytometry analysis at day 12 of differentiation of CD34^+^ cells and **(E)** CD31^+^ cells after NK5A induction with 0.5 or 1 of doxycycline starting at day 3 or day 5. **(F)** Representative dot plots analysis of hematopoietic progenitors (CD34^+^CD43^+^) and primitive hematopoietic cells (CD34^−^CD43^+^) at day 12 after NK5A induction with doxycycline starting at day 5 of differentiation. **(G)** Flow cytometry analysis of hematopoietic progenitors (CD34^+^CD43^+^) and **(H)** primitive hematopoietic cells (CD34^−^CD43^+^) at day 12 after NK5A induction with 0.5 or 1 μg/ml of doxycycline starting at day 3 or day 5. **(I)** RT-PCR analysis of HOXA5, HOXA6, HOXA7, HOXA9, HOXA10, and MEIS1 expression on cells floating in the culture supernatant at day 12 after NK5A induction with 0.5 or 1 μg/ml of doxycycline starting at day 3 or day 5. GAPDH, rRNA 18S, RPL7 and B2M were used as housekeeping control genes. All data was normalized by H9 WT—Dox. Undiff = Undifferentiated cells. Two-way ANOVA test applied in all comparisons. Significance; ** p-value < 0.01, *** p-value < 0.001 and **** p-value < 0.0001.

Increasing evidence support that most pediatric leukemias originate by the acquisition of driving genetic alterations before birth (Greaves et al., 2003; Cazzola et al., 2021). However, it is unknown the developmental stage and identity of the cell(s) in which the genetic hit occurs (Cazzola et al., 2021; Lopez et al., 2019). Thus, we decided to induce the expression of NK5A during the hematopoietic differentiation of the H9 iNK5A clonal lines using the STEMdiff™ Hematopoietic Kit (Stem Cell Technologies) (Figure 2A). First, we assessed the modulation of NK5A expression at different doxycycline concentrations (0.5, 1, and 2 μg/ml) from differentiation day 3 onwards. As expected, the amount of NK5A induction depended on the dosage of doxycycline (Figure 2B), however, at 2 μg/ml of doxycycline the NK5A expression level had a negative impact on cell viability (Figure 2C), and hence we decided to exclude this condition from our analysis. In contrast, the addition of doxycycline did not produce any effect on the H9 WT cells (Figures 2C–H). At day 12, we analyzed the hematopoietic differentiation of the H9 WT and H9iNK5A clonal cell lines after treatment with 0.5 and 1 μg/ml of doxycycline from day 3 onwards, when the mesoderm is induced, and from day 5 onwards, when the first hemato-endothelial progenitors (CD31^+^CD34^+^CD43^−^) are generated (Husa et al., 2018). By flow cytometry we observed in both H9 iNK5A clones that NK5A induction at day 3 strongly abolished the production of CD34^+^ and CD31^+^ cells (Figures 2D,E), and hematopoietic progenitors CD34^+^CD43^+^ (Figure 2G), and hardly produced primitive hematopoietic cells CD34^−^CD43^+^ (Figure 2H). In contrast, NK5A induction at day 5 only affected the generation of CD43^+^ primitive hematopoietic cells in both H9 iNK5A clones (Figures 2D–H). To investigate the effect of NK5A induction at transcriptional level we analyzed by RT-PCR the expression of known NK5A-targets genes (Wang et al., 2009). Our preliminary results show that the induction of the fusion protein enhanced the expression of the A-cluster HOX genes but did not affect the expression of MEIS1 (Figure 2J). Moreover, we observed higher expression levels of the HOX A genes when inducing NK5A earlier (day 3 vs. day 5) during hematopoietic differentiation (Figure 2J).

## Conclusion

Human ESCs and their derivatives resemble embryonic or fetal stages of human development; therefore, the inducible control of an oncoprotein expression in hESC brings the possibility of mimicking the malignant transforming events occurring in the embryonic development in pediatric leukemia. Using defined and controlled genomic tools we generated two H9 clonal cell lines with inducible expression of NUP98-KDM5A that maintain the pluripotent phenotype. There was a low-level of leakiness in the absence of doxycycline that was insignificant when compared with induced levels of NK5A RNA after the addition of the drug; and also, it was undetectable at protein level. In addition, the hematopoietic differentiation potential of H9 iNK5A#5 and #16 clones in the absence of doxycycline was similar to the H9 WT cells, demonstrating that the leaking levels of the fusion gene RNA are not enough to produce a phenotype.

Here we proved that the early induction of NUP98-KDM5A expression during the EB differentiation provoked several changes in cell differentiation. Also, we have shown that expressing the fusion protein at different stages during hematopoietic differentiation had a different effect on the production of the hematopoietic progenitors and their further differentiation. Experiments in mouse models suggest that the ontogeny of the cell targeted by a fusion oncogene affect the phenotype, latency and aggressiveness of the leukemia (Stavropoulou et al., 2016; Lopez et al., 2019; Sinha et al., 2020). NUP98-KDM5A fusion is most frequently found in acute megakaryoblastic leukemia, but it has also been detected in other AML subtypes (Noort et al., 2021) and it is possible that the cell subtype in which the genetic hit takes place determine the resulting AML phenotype, as has been demonstrated for the ETO-GLIS2 fusion protein (Lopez et al., 2019). Therefore, we are performing a more detail characterization of this model to determine which cell population is more susceptible for malignant transformation and how the cellular origin impacts on the leukemogenesis processes.

Finally, the same cellular system can be used for other types of pediatric malignancies where the identification of the cellular origin is still unrevealed.

## Data Availability

The original contributions presented in the study are included in the article/Supplementary Material, further inquiries can be directed to the corresponding author.

## Supplementary Material

Supplementary Figure S1
**(A)** Quantification of vector copy numbers by RT-PCR in H9 iNK5A bulk and H9 iNK5A #5 and #16 clones. H9 WT was used as negative control. **(B)** RT-PCR analysis of NUP98-KDM5A expression in the H9 iNK5A clonal lines grown in the presence or absence of 2 μg/ml of doxycycline for 48 h. GAPDH was used as housekeeping control gene. **(C)** Western blot analysis of H9 iNK5A#5 and #16 clonal lines after doxycycline treatment to detect NUP98-KDM5A fusion protein with the anti-KDM5A antibody. We used ACTIN as a loading control. **(D)** Expression of the pluripotency markers POU5F1, SOX2, NANOG and REX1 by RT-PCR. GAPDH was used as housekeeping control. All data was normalized by H9 WT. **(E)** Short Tandem Repeat (STR) profiling of H9 iNK5A#5 and #16 transgenic clonal lines with the original H9 cell line **(F)**. Karyotyping analysis of H9 iNK5A #5 and #16 clones showing a normal female 46, XX composition. **(G)** Western blot of the EBs differentiated for 21 days from H9 WT, iNK5A#5 and #16 clonal cell lines in presence or absence of 2 μg/mL of doxycycline. The induction of NK5A is detected with the antibody anti-FLAG and the ACTIN is used as a loading control. **(H)** Pictures of embryoid bodies (EBs) differentiated for 21 days from H9 WT, iNK5A#5 and #16 clonal cell lines with (+DOX) or without (−DOX) doxycycline treatment. Scale bar = 1000 μm.
