# Supplementary figures and images for: Generation of a H9 Clonal Cell Line With Inducible Expression of NUP98-KDM5A Fusion Gene in the AAVS1 Safe Harbor Locus
Source: Front Cell Dev Biol. 2022 Jun 1;10:846092. doi: 10.3389/fcell.2022.846092 (PMC9200071; doi:10.3389/fcell.2022.846092)

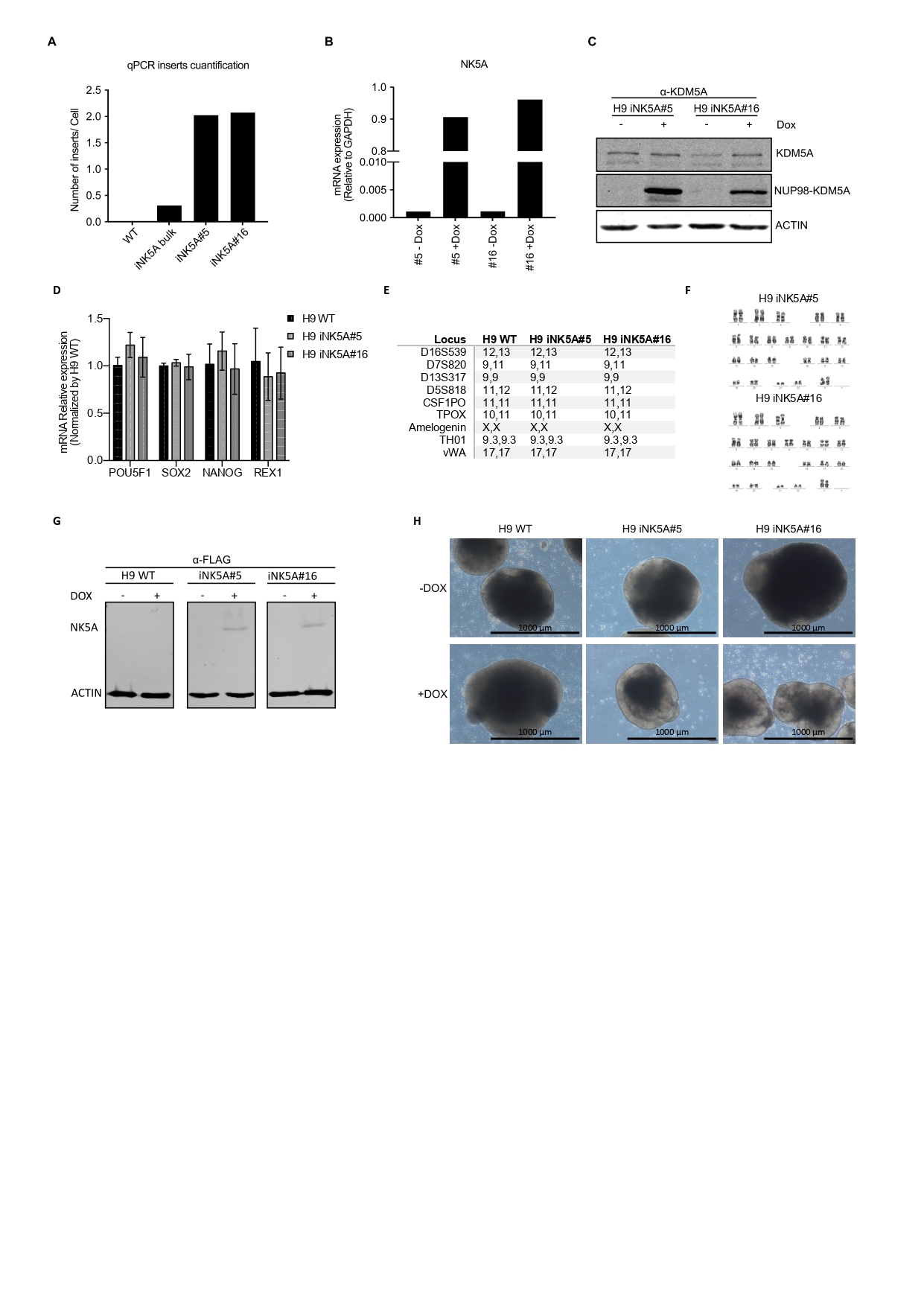

Supplement: Supplementary file 1 [file Image1.JPEG]
